# The effect of early postnatal discharge from hospital for women and infants: a systematic review protocol

**DOI:** 10.1186/s13643-016-0193-9

**Published:** 2016-02-08

**Authors:** Eleanor Jones, Beck Taylor, Christine MacArthur, Ruth Pritchett, Carole Cummins

**Affiliations:** Institute for Applied Health Research, University of Birmingham, Edgbaston, Birmingham, B15 2TT UK

**Keywords:** Postnatal care, Early discharge, Length of stay

## Abstract

**Background:**

The length of postnatal hospital stay has declined over the last 40 years. There is little evidence to support a policy of early discharge following birth, and there is some concern about whether early discharge of mothers and babies is safe. The Cochrane review on the effects of early discharge from hospital only included randomised controlled trials (RCTs) which are problematic in this area, and a systematic review including other study designs is required. The aim of this broader systematic review is to determine possible effects of a policy of early postnatal discharge on important maternal and infant health-related outcomes.

**Methods/design:**

A systematic search of published literature will be conducted for randomised controlled trials, non-randomised controlled trials (NRCTs), controlled before-after studies (CBA), and interrupted time series studies (ITS) that report on the effect of a policy of early postnatal discharge from hospital. Databases including Cochrane CENTRAL, MEDLINE, EMBASE, CINAHL and Science Citation Index will be searched for relevant material. Reference lists of articles will also be searched in addition to searches to identify grey literature. Screening of identified articles and data extraction will be conducted in duplicate and independently. Methodological quality of the included studies will be assessed using the Effective Practice and Organisation of Care (EPOC) criteria for risk of bias tool. Discrepancies will be resolved by consensus or by consulting a third author. Meta-analysis using a random effects model will be used to combine data. Where significant heterogeneity is present, data will be combined in a narrative synthesis. The findings will be reported according to the preferred reporting items for systematic reviews (PRISMA) statement.

**Discussion:**

Information on the effects of early postnatal discharge from hospital will be important for policy makers and clinicians providing maternity care. This review will also identify any gaps in the current literature on this topic and provide direction for future research in this area of study.

**Systematic review registration:**

PROSPERO CRD42015020545

**Electronic supplementary material:**

The online version of this article (doi:10.1186/s13643-016-0193-9) contains supplementary material, which is available to authorized users.

## Background

The average length of postnatal stay in England has decreased over the last 40 years. In 1975, 32 % of women were discharged within 3 days of giving birth compared to 91 % of women in 2013–2014 [1, 2]. The proportion of women and babies who are discharged on the date of delivery has also increased. In 2005–2006, 16.5 % of women were discharged on the same day that they gave birth compared to 20.3 % of women in 2013–2014 [2]. Despite an increase in medical intervention during childbirth and more complex needs of women who become pregnant, there is some evidence to suggest that low-risk women and babies are being discharged from 4–6 h following birth [3, 4]. The decline in postnatal stay in hospital is consistent with the USA, Australia and Canada and is considered to be primarily policy driven in efforts to accommodate a rising birth rate [5, 6].

There is little data and some concern about whether early discharge of mothers and babies is safe. It has been suggested that early discharge from hospital leaves insufficient time for women and babies to establish breastfeeding and, as a result, leads to feeding-related problems [7]. In addition, it is argued that early discharge may increase the delay in the identification and treatment of maternal and infant morbidity [8, 9]. In contrast, others have suggested that early discharge from hospital creates opportunities for family-centred care, creates greater opportunities for families to bond in their home environment and is a safe and cost-effective way to provide postnatal care [10, 11].

The existing evidence on the effects of early postnatal discharge from hospital is inconclusive. The most recent Cochrane systematic review including 10 randomised controlled trials (RCTs) (involving 4489 women) compared early postnatal discharge with a standard length of stay. The pooled estimate of the included trials showed no statistically significant difference between early discharge and standard length of stay for infant readmission to hospital (relative risk (RR) 1.29 95 % CI 0.60–2.79) or other important outcomes [5]. One of the main limitations of this review is the methodological and clinical heterogeneity within included studies.

Firstly, the review authors used the definition of ‘early discharge’ given by each trial team, and these ranged from 12 h to 3.5 days postpartum [12–14]. As a result, early discharge in one trial was the equivalent of standard length of stay in another trial. Secondly, the definition of ‘healthy women and infants’ differed among trials where some studies excluded women with comorbidities such as diabetes and others did not [12, 13]. Finally, the trials had different co-interventions in the early discharge groups ranging from being monitored at home for the first 24 h after birth [15] to only having two home visits once discharged from hospital [12]. Statistical heterogeneity was found when data from the trials were pooled in meta-analysis, and this is likely due to the varying definitions of early discharge, differing co-interventions and populations which were not clinically comparable. As a result, it is difficult to draw meaningful conclusions about the impact of shortened or ‘early’ postnatal stay in hospital.

To look more specifically at the RCTs included in the existing systematic review, one RCT which included 2324 women found that infants were twice as likely to be readmitted to hospital in the first month postnatally if they were discharged early (<48 h) compared to a standard length of stay in hospital (>48 h) (RR 2.14 95 % CI 1.2–7.5). Although this trial is the largest of its kind, its validity and reliability were compromised by non-compliance in the allocated intervention (50 % non-compliance in the intervention group), poor recruitment (only 20 % of women eligible chose to take part) and a sample size which was not large enough to detect significant differences between the intervention and comparison groups. Other trials had similar methodological constraints [16–19]. Although an RCT is generally the best method to evaluate the effects of an intervention, in the context of evaluating early postnatal discharge from hospital, an RCT design is likely to be both problematic and impractical. This has discouraged researchers from conducting further RCTs to assess the effect of early discharge from hospital on infant or maternal morbidity.

To this end, several large retrospective cohort studies have been conducted looking specifically at maternal and infant readmission rates to hospital within 28 days of birth. More specifically, researchers have assessed infant readmission to hospital for jaundice, gastroenteritis, dehydration and poor weight gain [8, 20–23]. Researchers examining maternal readmission rates have looked specifically at readmissions for postpartum haemorrhage, retained products of conception, infection and postpartum psychosis [24, 25]. These causes of readmission are particularly relevant in exploring the effect of postnatal length of stay in hospital. It is suggested by some that these causes of readmission may reflect an inadequate assessment of readiness for discharge from hospital and could possibly be avoided if sufficient support is available in the early postnatal period [5, 8, 12]. However, several of these studies were conducted using routine hospital data and from healthcare insurance claims data and not all known confounding factors were measured or adjusted for in data analysis. It is not possible to infer any causal relationship between early discharge and infant morbidity using these types of studies alone.

Despite the existing literature available on early postnatal discharge from hospital, there is insufficient evidence to inform policy. Although there is an existing Cochrane review with clearly specified outcome measures [5], it is limited by significant clinical and methodological heterogeneity. In addition, it is clear that an RCT design in this context is compromised by poor recruitment and participant crossover. Taking this into consideration, this systematic review will address the same research questions and use the same objectives and outcome measures as the Cochrane review but will broaden the study design criteria to include both RCTs and quasi-experimental studies. In addition, to allow meaningful comparison across studies, this systematic review will further describe the clinical characteristics and discharge criteria for the women and infants included in primary studies and will explore the effect of clinical variation in subgroup analysis. Early discharge will be defined as <48 h to reflect contemporary postnatal discharge practices [2]. To ensure rigour, the protocol for this systematic review has been guided by the PRISMA P checklist (Additional file 1).

The aim of this systematic review is to determine the effects of a policy of early postnatal discharge (<48 h) for women and infants. It will consider whether there is an association between early postnatal discharge and readmission to hospital. It is hypothesised that early postnatal discharge may increase maternal and infant utilisation of health services.

As guided by the Cochrane review [5], the primary objective of this systematic review is to assess how effective an early postnatal discharge policy is in terms of important maternal and infant health related outcomes.

Specific objectives are to identify whether a policy of early discharge is associated with:

- infant readmission to hospital;
- duration of infant readmission;
- attendance at hospital emergency departments for infant health issues;
- the number of contacts with health professionals regarding infant health issues postdischarge;
- maternal readmission to hospital;
- duration of maternal readmission;
- attendance at hospital emergency departments for maternal health issues;
- the number of contacts with healthcare professionals regarding maternal issues post discharge;
- maternal depression, anxiety and fatigue after the birth;
- occurrence of breastfeeding problems and/or duration of breastfeeding .

## Methods/design

### Criteria for considering studies for the review

#### Types of studies

Included studies must be either a RCT, non-randomised controlled trial (NRCT), controlled before-after study (CBA) or interrupted time series study (ITS). As guided by Effective Practice and Organisation of Care group (EPOC), all RCTs and non-randomised control trials must have at least two intervention and control sites [26]. All interrupted times series studies must have a clearly defined point in time when the early discharge policy occurred and a minimum of three data points before and three after the intervention. Because terminology used to describe study designs can be ambiguous, the EPOC study design algorithm will be used to help determine the study design [26].

If there is a paucity of the studies described above, a supplementary review will also include good-quality cohort studies located at a single site.

#### Types of participants

Women and infants who are considered 'fit for discharge' by their healthcare practitioners. Women may have given birth in a consultant led unit, co-located midwife led unit or stand-alone midwife led unit. It is recognised that there will be considerable variation in how ‘fit for discharge’ is defined and this will be explored in subgroup analysis.

#### Types of interventions

A policy of early discharge from hospital where ‘early discharge’ refers to discharge that is <48 h following birth and earlier than standard care in the setting in which the intervention is implemented.

#### Types of outcome measures

Maternal and infant outcome measures will be guided by the Cochrane review [5].

#### Primary infant outcomes

- Proportion of infants readmitted to hospital within 7 days and within 28 days after birth

#### Secondary infant outcomes

- Proportion of infants readmitted for conditions which may be considered avoidable (including jaundice, dehydration, poor weight gain, gastroenteritis) in the first 28 days after birth
- Duration of infant readmission for infants admitted within 7 and within 28 days after birth
- Total duration of infant hospitalisation over the first 28 days
- Proportion of infants attending accident and emergency department within 7 days and within 28 days after the birth
- Proportion of infants seen by a health professional in a primary care setting for a health related problem in the first 28 days after birth
- Number of contacts with health professionals regarding the infant within 28 days after birth

#### Primary maternal outcomes

- Proportion of women readmitted for complications related to childbirth (including postpartum haemorrhage, retained products of conception, infection, postpartum psychosis) in the first 6 weeks after birth
- Proportion of women breastfeeding (exclusively or partially) at 48 h, 6 weeks and 6 months after birth
- Proportion of women scoring above the cut off score indicating probable depression on a validated standardized instrument for measuring depression

#### Secondary maternal outcomes

- Duration of readmission for women readmitted after birth
- Total duration of maternal readmission hospitalisation
- Proportion of women attending hospital accident and emergency department
- Number of contacts with health professionals regarding maternal health issues within 4 weeks after birth
- Proportion of women reporting infant feeding problems within 4 weeks after birth

### Methods for identification of studies

#### Electronic searches

The following electronic databases will be searched:

- CENTRAL (Cochrane Central Register of Controlled Trials)
- MEDLINE
- EMBASE
- CINAHL
- Science Citation Index

Searches for relevant literature on these databases will be done using a combination of free text and indexed terms (for example MeSH terms) and will be combined using Boolean operators. Search terms will be adjusted for each electronic database. Due to wide variation in the definitions of study designs, the search strategy will not be limited by study design type.

### Proposed search strategy

Database: Ovid MEDLINE:

1. exp postnatal care/
2. postnatal.ti,ab.
3. postpartum period/
4. puerperium.ti,ab.
5. puerperium/
6. postpartum.ti,ab.
7. "length of stay"/
8. patient discharge/
9. discharge.ti,ab.
10. hospital stay*.ti,ab.
11. (early adj3 discharge).ti,ab.
12. patient readmission/
13. readmission*.ti,ab.
14. admission*.ti,ab.
15. hospitalization/
16. outcome*.ti,ab.
17. hospitali*.ti,ab.
18. safety.ti,ab.
19. complication*.ti,ab.
20. patient admission/
21. 1 or 2 or 3 or 4 or 5 or 6
22. 7 or 8 or 9 or 10 or 11
23. 12 or 13 or 14 or 15 or 16 or 17 or 18 or 19 or 20
24. 21 and 22 and 23

### Citation searching

Citation searching using key papers which meet the inclusion criteria for the review will enable identification of further potentially eligible studies.

### Grey literature

Grey literature will also be searched using Popline, Trip database and Web of Science conference proceedings citation index. The Department of Health, Royal College of Midwifery, Royal college of Obstetricians and Gynaecologists, National Childbirth trust and electronic theses (EThOS) websites will also be checked for relevant material. Internet searches will be performed in Google for any relevant unpublished studies.

No time, language or geographical restrictions will be applied.

### Searching other resources

Reference lists of key full text articles included in the review will be checked to identify any potentially eligible studies.

### Data collection and analysis

#### Selection of studies

All studies identified using the search strategy described will be screened for inclusion in the review using the eligibility criteria specifically designed to answer the research questions. Initially, a decision for potential inclusion of study will be based on titles and abstracts and where there is uncertainty about whether a study meets the eligibility criteria, over caution will be applied and the full article will be obtained for detailed assessment against the inclusion criteria.

Assessment of studies for potential inclusion will be performed independently and unblinded by two reviewers without consideration for the results. Any differences in opinions will be resolved through discussion until a consensus is reached. Any papers that are not unanimously excluded or included by both reviewers will be examined by both reviewers until an outcome is agreed. If necessary, a third person may be consulted. This process will ensure that bias is minimised when deciding whether to include or exclude certain studies.

It is possible that the eligibility criteria may need to be adjusted if it becomes apparent that relevant studies are being excluded from the review or irrelevant studies are being included. Reference management software EndNote will be used by one reviewer to keep a record of decisions made for each article in addition to paper form.

If there is a lack of information about a particular study, the authors will be contacted for clarification. Duplicate publications of research results will be identified and treated as a single study for the purpose of the review. In order to maintain transparency in the review study selection process, a flow diagram will portray the number of studies remaining in each stage of the selection process. In addition, a list of the studies excluded from the review will be documented as an appendix with brief reasons for exclusion for studies.

### Data extraction

As guided by University of York Centre for Reviews and Dissemination [27], data extraction will be performed independently by two reviewers and disagreements will be noted and resolved by consensus among researchers or by arbitration by a third researcher. The EPOC data collection form has been adapted to answer the specific research questions for the systematic review. The form will enable data collection of potential confounding factors in primary studies and methods used to control confounding.

In line with good practice, data extraction forms will be piloted on a sample of included studies to ensure that all the relevant information is captured and that resources are not wasted on extracting data that is not required [27].

### Assessment of methodological quality of included studies

It is likely that the methodological rigour of the included studies for this review will vary considerably, and it is recognised that that poor-quality studies may obscure important intervention effects or lack of effects. Due to the inclusion of quasi-experimental studies which may be more susceptible to bias than RCTs, thorough assessment of the study quality will be required.

Two review authors will independently assess the risk of bias of the included studies using a descriptive approach and guided by the EPOC criteria for ‘risk of bias’ tool. Risk of bias for each study included in the review will be qualitatively summarised as part of the summary of findings tables.

The quality assessment tool will be piloted on a small selection of included studies. It is recognised that the quality assessment of studies may involve a degree of subjective judgement and any differences in opinion will be resolved through discussion.

In addition to this, if a supplementary review which includes good-quality cohort studies is necessary, the Strengthening the Reporting of Observational Studies in Epidemiology (STROBE) checklist for cohort studies [28] will be used as a quality assessment tool and risk of bias will be qualitatively summarised in a risk of bias table.

### Measures of intervention effect

For RCTs, quasi-RCTs and CBAs, categorical data (e.g. proportion of infants readmitted) will be reported as RRs with 95 % confidence intervals. Continuous data (e.g. duration of infant hospitalisations in the first 28 days) will be reported as mean difference (MD) and 95 % confidence intervals. To aid the interpretation of clinical significance, the absolute risk may also be reported. Guidance will be sought from a statistician if meta-analysis of continuous outcomes requires standardisation across studies.

For ITS, as guided by Ramsey et al. [29], two effect sizes will be reported including the change in the outcome immediately after the introduction of the intervention and the change in slope of the regression lines.

### Unit of analysis errors

#### Methods for reanalysis of RCT and CBAs with potential unit of analysis error

As guided by EPOC [30], cluster RCTs which have been analysed incorrectly by not accounting for clustering will be reanalysed if possible. If the unit of analysis error cannot be corrected, the effect size will be reported without a standard error and confidence interval as they are unlikely to be accurate.

#### Methods of reanalysis for ITS comparisons with inappropriate analysis

Where possible, as guided by EPOC, segmented time series regression will be used to reanalyse the data using methods described in Ramsay et al. [29].

### Dealing with missing data

In line with good practice, authors of primary studies will be contacted to obtain missing data in order to appropriately describe the study results or perform meta-analysis [28]. The potential impact of missing data will be addressed in sensitivity analysis.

### Assessment of heterogeneity

It is likely that there will be much diversity in the included studies, and the variety of study designs included in the review may result in significant heterogeneity. Forest plots will be visually examined and poor overlap between the confidence intervals will give an indication of statistical heterogeneity. The *χ*^2^ test of heterogeneity will help to determine whether differences between results are compatible with chance alone and the *I*^2^ statistic will describe the percentage of variability in the effect estimates that can be attributed to heterogeneity rather than chance [31]. Where meta-analysis has been conducted, heterogeneity will also be explored through subgroup analysis.

### Data synthesis

A summary of findings table of included studies will be produced for primary outcomes and will include key information concerning the type of study, number and characteristics of participants, interventions, outcomes and outcome measures.

Where appropriate, meta-analysis using a random effects model will be used to combine data. It is anticipated that there will be much diversity in the included studies and there will be considerable variation in what constitutes ‘early discharge’. Where it is not appropriate to perform meta-analysis, included studies will be combined in a narrative synthesis and the results of the included studies will be combined in a forest plot with omission of the pooled estimate. To ensure the synthesis is a rigorous and transparent process, a narrative synthesis framework produced by Rodgers et al. [32] will be used. High-quality evidence will be given priority and results that are highly prone to bias will be interpreted with great caution.

### Sensitivity analyses

Where meta-analysis is performed for primary outcomes, sensitivity analyses will explore the effect of

- study quality (by performing two meta-analyses, one which includes all eligible studies and another which only includes high-quality studies as defined by EPOC quality assessment criteria).
- missing data (by using a range of assumptions about the outcomes for participants lost to follow-up in the intervention versus the control arms varying from 100 % intervention participants having a poor outcome to 0 %).

These sensitivity analyses have been decided a priori; however, further sensitivity analyses may be conducted if necessary.

### Subgroup analyses

Subgroup analyses will investigate the effect of variables which may moderate the primary outcomes examined in the review and explore potential effect modifiers for primary outcomes. Using evidence from existing literature [5, 9, 24], the following subgroups have been identified:

- Parity (primiparous women versus multiparous women)
  - Gestation ( <37 weeks, 37–40 weeks, 40+ weeks)
  - Timing of early discharge (≤12 h, ≤24 h, ≤48 h)
  - Mode of delivery (vaginal delivery, operative vaginal delivery, elective caesarean, emergency caesarean)
  - Co-intervention: early discharge accompanied by co-interventions (antenatal preparation or not, midwife home visits or not)
  - Type of hospital delivered at (consultant led unit, co-located midwife led unit, stand-alone midwife led unit)
  - Clinical characteristics of participants (low-risk women/infants, high-risk women/infants)
  - ‘Fit for discharge’ criteria (for example, some primary studies may consider women who had blood loss of >500 ml or third degree perineal trauma as ineligible for early discharge and other studies may not).

## Discussion

There is little evidence to support a policy early discharge from hospital. This systematic review aims to build on the work of the existing Cochrane review [5] by further describing the participant inclusion criteria and utilising a wider range of study designs to determine the effects of early postnatal discharge from hospital for mothers and infants. To reflect current postnatal practices, this systematic review will also further define early discharge as <48 h after delivery. It is anticipated that this review will identify any gaps in the current literature on this topic and provide direction for future research in this area of study.

## Additional file

Additional file 1:
**PRISMA-P 2015 checklist: recommended items to include in a systematic review protocol.** Completed PRISMA-P 2015 checklist for systematic review protocol: the effects of early postnatal discharge for women and term infants. (DOCX 16 kb)

## Abbreviations

CBA: controlled before-after studies
EPOC: Effective Practice and Organisation of Care
ITS: interrupted time series
MD: mean difference
NRCTs: non-randomised controlled trials
RCT: randomised controlled trials
RR: risk ratio
